# DeepSpectrumLite: A Power-Efficient Transfer Learning Framework for Embedded Speech and Audio Processing From Decentralized Data

**DOI:** 10.3389/frai.2022.856232

**Published:** 2022-03-17

**Authors:** Shahin Amiriparian, Tobias Hübner, Vincent Karas, Maurice Gerczuk, Sandra Ottl, Björn W. Schuller

**Affiliations:** ^1^Chair of Embedded Intelligence for Health Care and Wellbeing, University of Augsburg, Augsburg, Germany; ^2^Group on Language, Audio, and Music (GLAM), Imperial College London, London, United Kingdom

**Keywords:** computational paralinguistics, audio processing, transfer learning, embedded devices, deep spectrum

## Abstract

Deep neural speech and audio processing systems have a large number of trainable parameters, a relatively complex architecture, and require a vast amount of training data and computational power. These constraints make it more challenging to integrate such systems into embedded devices and utilize them for real-time, real-world applications. We tackle these limitations by introducing DeepSpectrumLite, an open-source, lightweight transfer learning framework for on-device speech and audio recognition using pre-trained image Convolutional Neural Networks (CNNs). The framework creates and augments Mel spectrogram plots on the fly from raw audio signals which are then used to finetune specific pre-trained CNNs for the target classification task. Subsequently, the whole pipeline can be run in real-time with a mean inference lag of 242.0 ms when a DenseNet121 model is used on a consumer-grade *Motorola moto e7 plus* smartphone. DeepSpectrumLite operates decentralized, eliminating the need for data upload for further processing. We demonstrate the suitability of the proposed transfer learning approach for embedded audio signal processing by obtaining state-of-the-art results on a set of paralinguistic and general audio tasks, including speech and music emotion recognition, social signal processing, COVID-19 cough and COVID-19 speech analysis, and snore sound classification. We provide an extensive command-line interface for users and developers which is comprehensively documented and publicly available at https://github.com/DeepSpectrum/DeepSpectrumLite.

## 1. Introduction

Over the past decade, the number of wearable devices such as fitness trackers, smartphones, and smartwatches has increased remarkably (van Berkel et al., 2015). With a rising amount of sensors, these devices are capable of gathering a vast amount of users' personal information, such as state of health (Ko et al., 2010), speech, or physiological signals including skin conductance, skin temperature, and heart rate (Schuller et al., 2013). In order to automatically process such data and obtain robust data-driven features, deep representation learning approaches (Amiriparian et al., 2017b; Freitag et al., 2017) and end-to-end learning methodologies (Tzirakis et al., 2018) can be applied. These networks, however, have a large number of trainable parameters (correlated with the large model size) and need a high amount of data to achieve a good degree of generalization (Zhao et al., 2019). These factors increase the energy consumption of the trained models (Yang et al., 2017) and confine their real-time capability. Furthermore, whilst personal data in unprecedented volumes is “in transit” or being synchronized with the cloud for further processing, it is susceptible to eavesdropping (Cilliers, 2020), and this issue raises privacy and security concerns for the user (e.g., discriminatory profiling, manipulative marketing; Montgomery et al., 2018). Such restrictions emerged the need for novel neural architectures and collaborative machine learning techniques without centralized training data (Li et al., 2020). Recent advancements include “squeezed” neural architectures (Iandola et al., 2016) and the federated learning paradigms (Li et al., 2020). Iandola et al. (2016) have introduced SqueezeNet, a “pruned” Convolutional Neural Network (CNN) architecture with 50× fewer trainable parameters than AlexNet (Krizhevsky et al., 2012) with an AlexNet-level accuracy. A more squeezed architecture, SqueezeNext, with 112× fewer parameters than AlexNet (with similar accuracy) was introduced by Gholami et al. (2018). Mehta et al. (2019) have proposed ESPNetv2, a lightweight general-purpose CNN with point-wise and depth-wise dilated separable convolutions for representation learning from large receptive fields with fewer parameters. Further energy-efficient CNN architectures have been proposed and applied for traffic sign classification (Zhang et al., 2020) and optical flow estimation (Hui et al., 2018).

For effective utilization of deep CNNs and to cope with data scarcity in the field of audio signal processing, we have introduced the Deep Spectrum system^1^ (Amiriparian et al., 2017c) at INTERSPEECH 2017. In Amiriparian et al. (2017c), we have forwarded (Mel) spectrogram plots of audio signals with different color mappings through pre-trained CNNs and extracted the activations of the penultimate fully connected layer of these networks as a feature set. For the effect of different color maps on the representations, please refer to Amiriparian et al. (2017c, 2019, 2020). Deep Spectrum features have shown to be effective for a variety of paralinguistic and general audio recognition tasks, including Speech Emotion Recognition (SER) (Ottl et al., 2020), sentiment analysis (Amiriparian et al., 2017a), and acoustic surveillance (Amiriparian et al., 2018). Furthermore, the Deep Spectrum system has been proved to be a competitive baseline system for the 2018–2021 editions of the Computational Paralinguistics Challenge (ComParE) (Schuller et al., 2019, 2021). In this article, we propose DeepSpectrumLite, an extension of the Deep Spectrum framework for embedded speech and audio processing. Whereas, the Deep Spectrum framework extracts features from pre-trained CNNs, DeepSpectrumLite goes one step ahead. First, it adds a lightweight Multilayer Perceptron (MLP) to the neural network pipeline which is responsible for either classification or regression. Second, the DeepSpectrumLite offers support for efficient on-device computation of the audio signal processing including the generation and on-the-fly augmentation of spectrogram image plots. Further, it allows fine-tuning of the image CNNs for each audio recognition task. The proposed system implements a model and inference structure that is focused on mobile usage, thereby computationally expensive audio signals processing can be performed efficiently on embedded devices. We make our DeepSpectrumLite framework publicly available for users and developers on GitHub^2^ and PyPI.

The remainder of this article is organized as follows: in Section 2, we describe the architecture of the proposed system. Subsequently, we introduce the datasets applied for our experiments, outline the experimental settings and results, and analyse the explainability challenges in Section 3. Afterwards, in Section 4, we discuss the performance of the trained models and their inference time on embedded devices. Finally, concluding remarks and our future work plans are given in Section 5.

## 2. Proposed System

Our framework is composed of two main parts: (i) task-specific, transfer learning-based model training (cf. Section 2.1), and (ii) decentralized audio processing using the trained model (cf. Section 2.2). For the first component of DeepSpectrumLite, we make use of pre-trained neural networks (instead of training a new network from scratch) to achieve a better generalization for audio recognition tasks in which the data of the target class is scarce (Shie et al., 2015; Hutchinson et al., 2017; Amiriparian, 2019). In the second component of the framework, the pre-trained (and fine-tuned) model is adapted to be run on embedded devices. In the developed architecture, both components perform in synchrony facilitating low-resource signal processing for a variety of speech and audio tasks.

### 2.1. Task-Specific Transfer Learning

The input of our system consists of raw audio signals with a sample rate of 16 kHz. For simplicity, our system reads only one audio channel. Subsequently, we apply a sliding window function to split the audio signals into smaller fixed-width chunks. For each chunk, we apply a signal normalization and then a Short-Time Fourier Transform (STFT) with Hanning windows of 32 ms and 50.0% hop length. The spectrograms are then transformed into Mel spectrograms with 128 Mel bins. We further compute the power spectral density on the *dB* power scale and apply a *min-max normalization* which is linearly scaled between [0, 255]. Subsequently, each value in the rescaled spectrogram matrix is mapped according to the *viridis* color definition. Since we use image CNNs that were pre-trained on ImageNet (Deng et al., 2009; Huang et al., 2017), we resize the spectrogram image plot to 224 × 224 pixels with bi-linear interpolation and mean normalize the image color channel values according to the original ImageNet dataset. Afterwards, we use the deep CNN model DenseNet121 (Huang et al., 2017) as a convolutional feature extractor for the generated audio plot images and attach an MLP classifier containing a single hidden layer with Attention-based Rectified Linear Unit (AReLU) (Chen et al., 2020) activation on top of this base. To reduce the effect of overfitting, we further apply the regularization technique dropout (Srivastava et al., 2014).

We have used DenseNet121 with weights pre-trained on ImageNet data as the feature extractor for two main reasons. First, we want to directly compare our results for the COVID-19 Cough (CCS), COVID-19 Speech (CSS), Escalation at Service-desks and in Trains (ESS), and Interactive Emotional Dyadic Motion Capture (IEMOCAP) corpora to the official evaluation setting of the ComParE 2021 Challenge (Schuller et al., 2021). Second, Amiriparian et al. (2020) has shown that an image pre-trained DenseNet121 is superior to other CNNs (in particular ResNet50, VGG16, VGG19) with pre-trained and random weights.

The training of our transfer learning models then proceeds in two phases. In the first phase, we freeze the CNN model structure and only train the classifier head. In the second phase, we unfreeze a part of the CNN's layers and continue training with a reduced learning rate. Furthermore, we apply different data augmentation techniques to the spectrogram plots on the fly during training. Data augmentation helps to reduce the effect of overfitting, especially when only a small number of training samples is available (Perez and Wang, 2017; Shorten and Khoshgoftaar, 2019).

DeepSpectrumLite has implemented an adapted version of the SapAugment data augmentation policy (Hu et al., 2021). The policy decides for every training sample its portion of applied data augmentation. We apply both CutMix (Yun et al., 2019) and SpecAugment (Park et al., 2019) data augmentations relatively to the loss value of all samples within a batch. The basic idea of SapAugment is that a training sample with a comparably low loss value is easy to understand using the current weights of a neural network, therefore, more data augmentation can be applied. Whereas, when a sample has a comparably high loss, SapAugment argues that less data augmentation should be applied until the sample reaches a low loss value. For further details on how the portion of applied data augmentation relative to the loss value is computed, the interested reader is referred to Yun et al. (2019).

### 2.2. Decentralized Audio Processing

After centralized training of a task-specific model, its network structure and weights are saved into a Hierarchical Data Format (HDF) version 5. The saved model is then converted to TensorFlow (TF) Lite^3^ format for compatibility on embedded devices.

**Figure 1:**
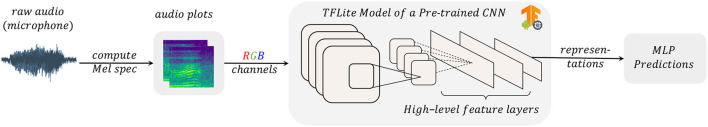
A general overview of a DeepSpectrumLite model deployed on a target device for inference. Raw audio (from the device's microphone) is first converted to a spectrogram representation and the values are mapped to the red-green-blue (RGB) color space according to a certain color mapping definition. These spectrogram plots are then forwarded through the TFLite version of a trained CNN model, and an MLP classifier head generates predictions for the task at hand.

Since our framework applies all necessary preprocessing steps within the data pipeline structure, there is no device-specific implementation required. A schematic overview of DeepSpectrumLite deployed on a target mobile device is depicted in Figure 1. From the input raw audio signals (e.g., signals captured from a microphone) Mel spectrogram plots are created which are then forwarded through a TF Lite version of the model trained as described in Section 2.1. It consists of a (fine-tuned) image CNN, here a DenseNet121, and a lightweight MLP head that classifies the deep representations obtained from a specific layer of the CNN.

The whole **audio processing** steps during inference, including reading the raw audio signals (e.g., from the microphone of an embedded device), extraction of the features, and the classification are conducted in a decentralized way, i.e., removing the need to send the data to a server for processing and evaluation. By doing so, all users' data will remain on their smart devices. Therefore, we have refrained from utilizing methods such as federated learning for **model training**.

## 3. Experiments

We perform experiments regarding the general learning capabilities of DeepSpectrumLite by evaluating its efficacy on eight databases which are described briefly in Section 3.1. Our framework has a set of hyperparameters that are fine-tuned for each audio task (cf. Section 3.2). We further compare the performance of DeepSpectrumLite with the original Deep Spectrum system which showed state-of-the-art results for various audio recognition tasks (Zhao et al., 2018; Amiriparian et al., 2020). An MLP is used as the classifier in our experiments. Utilized hyperparameters for each experiment are provided in **Table 2**. We then investigate the suitability of the trained DeepSpectrumLite models for real-time audio classification on an embedded device (cf. Section 3.3).

### 3.1. Datasets and Partitions

We utilize a diverse set of datasets to cover a range of audio processing tasks from paralinguistics to digital health. For the task of SER and social signal processing, the Database of Elicited Mood in Speech (DEMoS) (Parada-Cabaleiro et al., 2020), IEMOCAP (Busso et al., 2008), and ESS (Schuller et al., 2021) are used. The datasets CCS, CSS, Munich-Passau Snore Sound Corpus (MPSSC), and Düsseldorf Sleepy Language Corpus (SLEEP) are applied for the audio-based digital health tasks. We further analyse the performance of our framework for music emotion recognition by using the Ryerson Audio-Visual Database of Emotional Speech and Song (RAVDESS) (Livingstone and Russo, 2018) dataset. All datasets are speaker-independently split into training, validation, and test partitions. This partitioning strategy is maintained for all experiments on all datasets in this manuscript. Detailed statistics about each dataset are provided in Table 1.

**Table 1:** Statistics of the databases utilized in our experiments in terms of number of samples (#), number of speakers (*Sp*.), number of classes (*C*.), total duration (*Dur*.) in minutes, and mean and standard deviation of the duration (*Mean & Std dur*.) in seconds.

**Name**	**#**	**C**.	**Sp**.	**Dur. [min.]**	**Mean dur. [s]**	**Std dur. [s]**
**CCS**: COVID-19 cough	725	2	397	97.8	6.34	2.2679
**CSS**: COVID-19 speech	893	2	366	194.4	13.16	5.4784
**DEMoS**: Elicited mood in speech	9,365	7	65	445.6	2.86	1.2564
**ESS**: Escalation in speech	914	3	21	58.7	3.86	1.6418
**IEMOCAP**: Emotional speech	5,531	4	10	746.2	4.46	3.0645
**MPSSC**: Snoring sounds	828	4	219	20.8	1.51	0.3464
**RAVDESS**: Emotional song	1,012	6	23	78.4	4.65	0.4213
**SLEEP**: Sleepiness prediction from speech	16,462	2	915	1059.8	3.86	0.6399

#### 3.1.1. CCS and CSS

The CCS and CSS datasets both deal with voice-based COVID-19 detection and were introduced as part of ComParE 2021 (Schuller et al., 2021). The CCS dataset consists of crowd-sourced audio samples of coughing, recorded from 397 subjects resulting in 725 audio clips. The CSS dataset contains 893 audio samples from 366 subjects. A preceding COVID-19 test of the subjects was *positive* for one part and *negative* for the rest. The result of this test should be predicted by the challenge participants based on the audio content. We use the official challenge partitions in our experiments.

#### 3.1.2. DEMoS

DEMoS is a corpus of induced emotional speech in Italian (Parada-Cabaleiro et al., 2020), including 9, 365 emotional and 332 neutral speech samples produced by 68 native speakers (23 females, 45 males) in seven emotional states (anger, disgust, fear, guilt, happiness, sadness, and surprise). The emotional speech in the dataset is elicited by combinations of Mood Induction Proceduress (MIPss) in order to obtain more realistic speech production in comparison to acted speech. A detailed description of DEMoS is given in Parada-Cabaleiro et al. (2020).

#### 3.1.3. ESS

The ESS corpus combines the dataset of aggression in trains (Lefter et al., 2013) and the stress at service desk dataset (Lefter et al., 2014). In total, 21 subjects were exposed to different scenarios that were recorded in 914 audio files. The original labels are mapped onto a 3-point scale, *low, medium*, and *high* escalation. The language in the clips is Dutch.

#### 3.1.4. IEMOCAP

The IEMOCAP dataset (Busso et al., 2008) is an English emotion dataset containing audio of (scripted and improvised) dialogues between 5 female and 5 male speakers, adding up to 5, 531 utterances. The chosen emotion classes are happiness (fused with excitement), sadness, anger, and neutral. The dataset is split into sessions 1–3 for training, session 4 for validation, and session 5 for testing. The splits' selection for the partitions is motivated by the EmoNet paper (Gerczuk et al., 2021) which thoroughly addresses the multi-corpus SER from a deep transfer learning perspective.

#### 3.1.5. MPSSC

The MPSSC (Janott et al., 2018) dataset includes audio recordings of 828 snore events from 219 subjects. These events are annotated in terms of the VOTE classification (V, velum; O, oropharyngeal lateral walls; T, tongue base; and E, epiglottis; Kezirian et al., 2011) for the location of snoring noise by experts. We use the official partitioning provided by the authors in the INTERSPEECH ComParE 2017 challenge (Schuller et al., 2017).

#### 3.1.6. RAVDESS

RAVDESS (Livingstone and Russo, 2018) is an audio-visual database containing emotional displays of both speech and song. For our experiments, we exclusively use the emotional song portion with 1, 012 samples recorded from 23 actors. The portrayed emotions are angry, calm, fearful, happy, and sad. The corpus has been extensively validated by 247 individuals, producing 10 ratings for every sample.

#### 3.1.7. SLEEP

We use a subset of the SLEEP Corpus created at the Institute of Psychophysiology, Düsseldorf that served as a ComParE challenge task for continuous sleepiness prediction in 2019 (Schuller et al., 2019). The set contains 16, 462 recordings, including reading passages, speaking tasks, and elicited spontaneous speech from 915 subjects (364 females, 551 males, between the ages of 12 and 84). The recordings are annotated in terms of the Karolinska Sleepiness Scale (KSS) (Shahid et al., 2011) (range 0 − 9) by averaging self-assessments and post-hoc observer ratings. For our experiments, we perform a binary discretization of our labels into *not sleepy* [0 − 7.5] and *sleepy* (7.5 − 9].

### 3.2. Hyperparameters

We train our models with the AdaDelta optimizer (Zeiler, 2012) on the cross-entropy loss function in batches of 32 samples. After training the classifier head for a certain number of initial epochs only, we reduce the learning rate 10-fold and continue training with some of the layers of the DenseNet121 unfrozen. As the datasets have different sizes and numbers of classes, we slightly adapt our hyperparameter configuration to each of them. All important details on the hyperparameters for each dataset are provided in Table 2. Furthermore, we evaluate four data augmentation configurations: (1) no augmentation, (2) only CutMix, (3) only SpecAugment, and (4) a combination of CutMix and SpecAugment.

**Table 2:** This table shows the configuration of the different hyperparameters for each of the datasets used in our experiments.

**Hyperparameter**	**CCS**	**CSS**	**DEMoS**	**ESS**	**IEMOCAP**	**MPSSC**	**RAVDESS**	**SLEEP**
Classifier units	512	700	512	512	512	512	128	512
Dropout rate	0.25	0.4	0.25	0.25	0.25	0.25	0.5	0.25
Initial learning rate	0.001	0.01	0.001	0.001	0.001	0.001	0.001	0.001
Epochs of first phase	40	20	40	40	40	40	40	40
Epochs of second phase	200	–	120	200	200	120	80	80
Fine-tuned layers	298	0	128	298	128	85	42	128
Audio chunk length [s]	3.0	–	3.0	3.0	4.0	1.0	4.0	4.0

In our experiments, we use SapAugment with the configuration values *a* = 0.5, *s* = 10. Our CutMix algorithm hyperparameters are set to cut and paste squared patches between sizes of [0*px*, 56*px*] among the training samples. The ground truth labels are proportionally mixed according to the pasted patch size. Moreover, the SpecAugment data augmentation creates a one-time mask and one frequency mask for every training sample. The size of every mask is between [0.0*px*, 67*px*]. The actual patch sizes and mask sizes depend on the samples' loss value (cf. Section 2.1). Because the number of available training samples is limited, we expect the problem of underfitting when applying data augmentation for every single training sample. Therefore, we throttle down the usage of all data augmentations by adding an execution probability between [10.0, 25.0%] dependent on the sample's loss value.

### 3.3. Results

We evaluate the performance on the test partitions using the Unweighted Average Recall (UAR) metric which is equivalent to balanced accuracy and provides more meaningful information when a dataset has an unbalanced class ratio. In Tables 3, 4, all results obtained *via* our framework with and without various augmentation techniques are provided. In Table 3, we show that our framework constantly outperforms the Deep Spectrum baselines of the ComParE 2021 Challenge by 16.1, 5.8, 9.4, and 6.0% relative improvement on the unseen test set for the CCS, CSS, ESS, and IEMOCAP (not part of ComParE) tasks, respectively. Furthermore, in order to be consistent with the ComParE methodology, for all datasets, we initially find the best set of hyperparameters by validating the trained model on the development set. Afterwards, we train a final model on the combined training and development partitions before evaluation on the test set. By doing so, we provide more data to our Deep Neural Networks (DNNs) and aim for better generalization capabilities.

**Table 3:** Results of the transfer learning experiments with DeepSpectrumLite (DS Lite) on three of the ComParE 2021 Challenge tasks (CCS, CSS, and ESS) and IEMOCAP compared against Deep Spectrum feature extraction + Support Vector Machine (SVM).

	**CCS**			**CSS**			**ESS**			**IEMOCAP**		
**[UAR %]**	**Dev**	**Test**	**CI on test**	**Dev**	**Test**	**CI on test**	**Dev**	**Test**	**CI on test**	**Dev**	**Test**	**CI on test**
Deep Spectrum + SVM (Schuller et al., 2021)	63.3	64.1	55.7−72.8	56.0	60.4	55.9−64.9	64.2	56.4	51.5−61.3	53.0	56.3	54.2−58.2
DS Lite (no augmentation)	56.5	71.1	62.2−79.5	61.6	61.2	55.1−66.8	43.1	60.0	54.3−66.1	55.1	56.4	55.2−63.9
DS Lite (CutMix)	57.1	71.4	62.5−79.4	62.2	62.3	56.4−68.4	43.1	59.9	54.1−65.7	55.5	**59.7**	55.6−64.0
DS Lite (SpecAugment)	58.4	72.7	63.9−80.9	60.7	63.6	58.1−69.0	48.1	61.3	55.4−67.2	55.2	59.3	54.9−63.5
DS Lite (CutMix+SpecAugment)	59.0	**74.4**	66.3−82.4	60.7	**63.9**	55.1−66.8	47.2	**61.7**	55.8−67.3	53.9	59.2	54.9−63.5

*For the ComParE tasks, we evaluate against the official Deep Spectrum results presented in Schuller et al. (2021) while for IEMOCAP, we run the Deep Spectrum challenge baseline with the same settings ourselves. CCS, COVID-19 cough; CSS, COVID-19 speech; ESS, escalation in speech; IEMOCAP, emotional speech; UAR, unweighted average recall; CI, 95.0% confidence interval. Chance level in UAR: 50.0, 50.0, 33.3, and 25.0% for CCS, CSS, ESS, and IEMOCAP, respectively. For each dataset, the best result on the test partition is highlighted in boldface*.

**Table 4:** Results of the transfer learning experiments with DeepSpectrumLite (DS Lite) on four datasets, the ComParE 2018 Snore Sub-Challenge tasks Snore, the ComParE 2019 Continuous Sleepiness Sub-Challenge SLEEP, RAVDESS emotional song and DEMoS.

	**MPSSC**			**SLEEP**			**RAVDESS**			**DEMoS**		
**[UAR %]**	**Dev**	**Test**	**CI on test**	**Dev**	**Test**	**CI on test**	**Dev**	**Test**	**CI on test**	**Dev**	**Test**	**CI on test**
DS Lite (no augmentation)	33.5	39.4	33.4−45.7	66.0	65.7	62.9−68.5	84.7	76.6	72.7−84.5	59.1	58.5	55.8−61.4
DS Lite (CutMix)	43.7	50.0	43.7−55.6	71.7	**69.1**	66.5−71.6	84.0	78.6	72.6−84.1	65.0	62.1	59.1−65.1
DS Lite (SpecAugment)	44.4	52.0	46.3−57.9	66.3	66.8	65.1−68.7	77.4	**81.3**	74.6−87.1	69.5	69.7	67.1−72.3
DS Lite (CutMix+SpecAugment)	39.2	**54.2**	49.4−58.4	67.6	67.6	64.9−70.4	71.8	75.0	68.9−80.5	73.8	**72.5**	70.1−75.0

*For the Sleep task, we discretize the label into two classes, while for the DEMoS task, we recreate the dataset partitioning used in Baird et al. (2019). SLEEP, sleepiness; MPSSC, snore sound; RAVDESS, emotional song; DEMoS, emotional speech; UAR, unweighted average recall; CI, 95.0% confidence interval; Chance level in UAR, 25.0, 50.0, 20.0, and 14.3% for MPSSC, SLEEP, RAVDESS, and DEMoS, respectively. For each dataset, the best result on the test partition is highlighted in boldface*.

For the other four datasets (cf. Table 4), a direct comparison with ComParE Challenges or similar baseline systems was not possible. However, for the sake of consistency, we follow the same partitioning and evaluation strategy across all experiments. For all eight datasets, it can be seen that the applied augmentation methods improve the overall performance compared to the experiments without any augmentation. This effect is more prominent when datasets are small (e.g., for CCS, CSS, ESS, and MPSSC). The highest impact can be seen for the MPSSC dataset for which the augmentation with CutMix+SpecAugment lead to a 37.6% relative improvement on the test set compared to the model without any augmentation. In five out of eight datasets, the fusion of the CutMix and SpecAugment method was demonstrated to be superior to using these augmentation methods individually. We additionally provide 95.0% Confidence Intervals (CIs) on the test partitions. They were obtained by 1, 000× bootstrapping. In each iteration, a random selection of test samples is replaced and the UAR is computed. Moreover, the unweighted chance level for each dataset is given in each result table.

### 3.4. Computational Performance

The number of Floating Point Operations (FLOPs) is used here as a measure of the efficiency of the introduced framework. The more FLOPs an algorithm needs to finish, the longer it takes to run and the more power it consumes. Embedded devices typically have a limited power capacity, as they have a battery and no continuous power supply. Therefore, we take the DeepSpectrumLite framework's power efficiency into account. This subsection examines the models' FLOPs, mean execution time, mean of requested memory, and the model size. Our analysis is split into the audio signal preprocessing step, i. e., the spectrogram plot creation, and the final model inference. Both the TensorFlow (TF) model, and the spectrogram creation were executed 50 times on a 2.3 GHz Quad-Core Intel Core i5 CPU with 4 threads. To investigate the difference to the TF Lite model, we tested both models on the same system. Furthermore, we examined an on-device test on a consumer-grade smartphone *Motorola moto e7 plus* which comes with a 4 × 1.8 GHz Kryo 240, a 4 × 1.6 GHz Kryo 240, and an Adreno 610 GPU. Every on-device test was repeated 50 times. Table 5 shows the performance results of our spectrogram image creation and the DenseNet121 model which includes the classification layers as well. The spectrogram creation has a model size of 150.0 kB, a mean execution time of 7.1 ms, and it consumes 4.5 MB memory. Because the plot generation is not a TF model, we cannot measure the FLOPs nor are there any parameters. However, the number of FLOPs is expected to be small based on the measured execution time. During the transformation from the TF HDF file format to the TF Lite model, the model size is reduced by the factor of 2.7. Although the TF Lite model consumes more memory than the regular TF model, the mean inference time is reduced by 150.3 ms measured on the same CPU setup. The TF Lite model has a mean inference time of 242.0 ms on our embedded device.

**Table 5:** This table shows the mean execution time, the number of parameters, the mean requested memory, and the model size of our preprocessing (prepr.), the DenseNet121 TensorFlow (TF), and TF Lite model.

	**Prepr**.	**TF model**	**TF lite model**
Mean time [ms]	7.1	240.0	89.7/242.0
FLOPs	–	3.1 G	–
Parameters	–	7.6 M	7.6 M
Mean memory [MB]	4.5	116.5	185.4/292.8
Model size	150.0 kb	82.1 MB	30.0 MB

*In the TF Lite Model column, the values before the slash are from the test on the CPU system, whereas the values after the slash are from the on-device test. Details regarding the test setup are described in the text. FLOPs, floating point operations*.

### 3.5. Explainability Challenges

The explainability of deep learning models is a well-known challenge that we would like to briefly mention in this part of our manuscript. For each component (audio processing, feature extraction, classification) of the DeepSpectrumLite framework, a different degree of explainability can be achieved. It should be mentioned that due to the complexity of the applied DNNs for deep feature extraction and classification, the obtained results and the behavior of the models cannot be thoroughly explained. However, we try to approximately explain the framework's decision-making process in a threefold manner. First, we provide Mel spectrogram plots for *negative* and *positive* classes of both CSS and CCS datasets as case examples and analyse them. Second, we discuss the DNN models' outputs with the help of SHAP (Lundberg and Lee, 2017) method. Third, we provide confusion matrices of the classification results and compare the confusion between class predictions.

**Figure 2:**
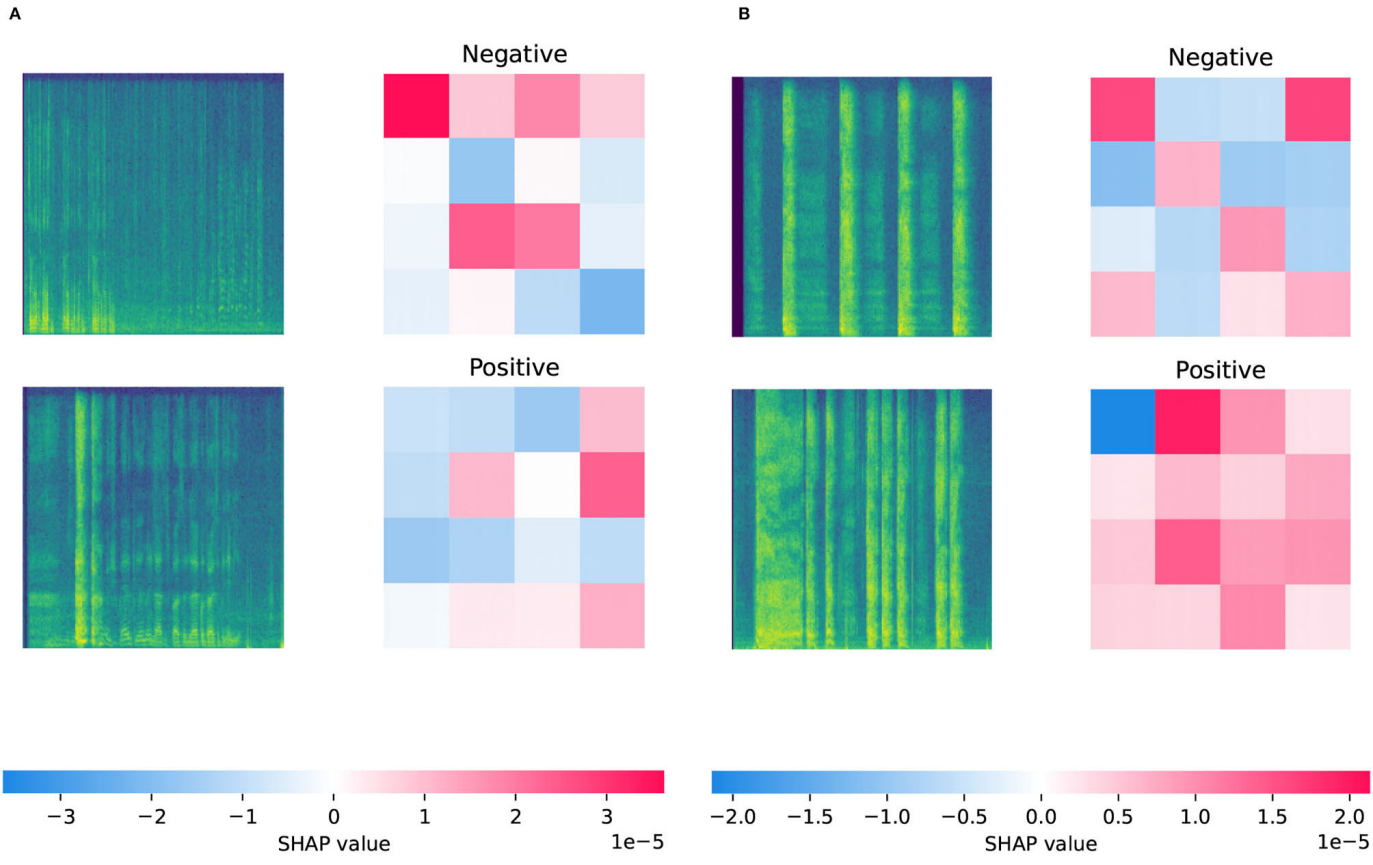
Visualization of the SHapley Additive exPlanations (SHAP) outputs for sample Mel spectrograms from *negative* and *positive* classes of CSS and CCS datasets. The x-axis of the spectrograms represents time [0–5 s] and the y-axis represents the frequency [0–4,096 Hz]. The range of the SHAP values for each model's output is given in the color bar below each image. Areas that increase the probability of the class are colored in red, and blue areas decrease the probability. A detailed account of the spectrogram and SHAP values analysis is given in Section 3.5. **(A)** SHAP outputs from the COVID-19 speech (CSS) model. **(B)** SHAP outputs from the COVID-19 cough (CCS) model.

For the first two parts of our explainability approach, we analyse sample Mel spectrograms from the *negative* and *positive* classes of the COVID-19 cough and speech datasets w. r. t. strength of the audio signals over time at a waveform's various frequencies. Further, we provide the outputs of SHAP (Lundberg and Lee, 2017), a game-theoretic approach to explain the output of machine learning models. SHAP assigns each feature—in our article, regions of the Mel spectrogram plots—an *importance value* for each particular class prediction (cf. right side of the Figure 2). The sample spectrograms of the *negative* class of both datasets show a harmonic broad-band pattern with a quite similar width. Contrarily, the *positive* class is characterized by disruptions in the amplitude for distinct frequency ranges, indicating discontinuities in the pulmonic airstream during phonation in COVID-19 *positive* participants (Bartl-Pokorny et al., 2021). The impact of each segment of the Mel spectrograms on the output of the DNN model is visualized with help of SHAP values. Areas of the audio plots that increase the probability of the class are colored in red, and blue areas decrease the probability. For example, for the given *negative* class sample of the CSS dataset, F0 or fundamental frequency (marked as blue squares on the bottom of the SHAP output) pushes the prediction for the *negative* class lower, whilst higher frequencies (marked as red squares on the top of the SHAP output) do the opposite. For the *positive* class sample of CSS, an almost reverse pattern can be seen. The more prominent the red squares, the higher the model's confidence in predicting the target class. For the COVID-19 cough samples, we see that the model is quite confident in predicting the *positive* COVID-19 cough, whilst for the *negative* class, mainly the F0 and top frequencies at the beginning and the end of the non-COVID-19 cough cause it to be classified as *negative*.

**Figure 3:**
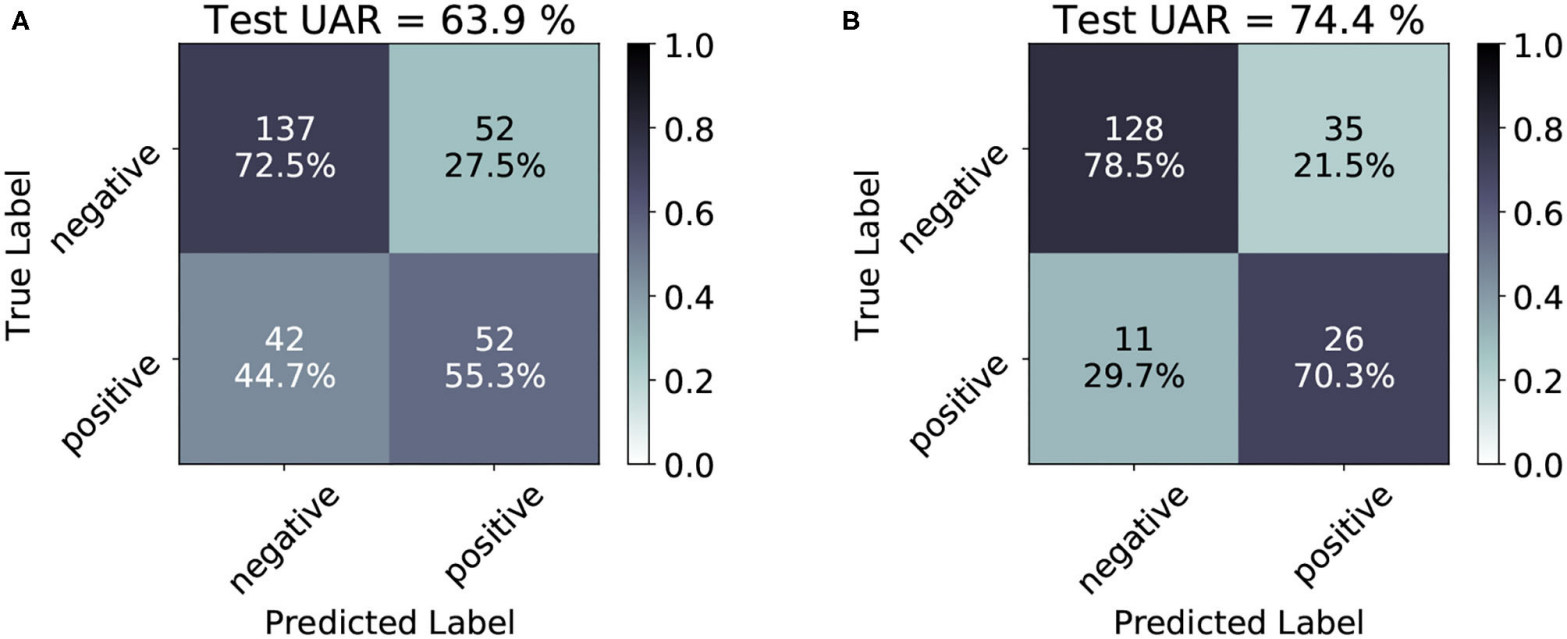
Confusion matrices for the test results obtained by the best CSS **(A)** and CCS **(B)** models. In the cells, the absolute number of cases is given, and the percentage of “classified as” of the class is provided in the respective row. The percentage values are also indicated by a color scale: the darker, the higher the prediction. A detailed account of the confusion matrices analysis is given in Section 3.5.

For the last part of our explainability approach, we analyse the confusion matrices of the classification results on the test partitions of CSS and CCS corpora. With the help of Figure 3, the True Positive Rate (TPR) (sensitivity), True Negative Rate (TNR) (specificity) and the class-wise performance of the DNN models for each task can be obtained. The specificity of the CSS model (72.5%) is much higher than its sensitivity (55.3%), indicating the model's ability to correctly reject healthy patients without COVID-19 and its challenges to correctly detect ill patients who do have COVID-19. On the contrary, the low confusion between the CCS classes compared to CSS implies the superiority of the ‘cough-based' model over the “speech-based” one for the recognition of COVID-19.

## 4. Discussion

The results achieved with DeepSpectrumLite (described in Section 3.3) on all eight tasks show the system's efficacy, in particular, compared to the traditional Deep Spectrum feature extraction. Furthermore, the applied state-of-the-art CutMix (Yun et al., 2019) and SpecAugment (Park et al., 2019) techniques in combination with an adapted version of the SapAugment (Hu et al., 2021) policy proved themselves to be useful for all datasets, especially for the smaller ones. For the IEMOCAP dataset, our best performing model achieves comparable results with the recently published EmoNet paper which uses the same partitioning strategy (Gerczuk et al., 2021).

A gap can be seen between the performance of the trained models on the development and test partitions. This could partially be explained by the lack of training materials for the MLP classifier while optimizing it on the development partition. On the other hand, the better performance on the test set could be a result of training the final model on the combined train and development sets before evaluating on the test partition. Further, the effect of dataset imbalance and suboptimal distribution of audio samples in each partition should not be neglected here.

Considering embedded devices, such as consumer-grade smartphones, as deployment targets, DeepSpectrumLite is further suitable for real-time speech recognition tasks. With a total inference time of only a quarter of a second for a 3-s long raw audio chunk, time-continuous analysis from raw microphone input can be performed directly on-device. The measured performance, both in terms of recognition accuracies on the datasets as well as inference times, make DeepSpectrumLite a powerful framework for many paralinguistic and general audio recognition tasks where data is often scarce.

## 5. Conclusion

In this article, we presented a framework for training and deploying power-efficient deep learning models for embedded speech and audio processing. By making use of transfer learning from ImageNet pre-trained deep CNNs with spectrogram inputs and state-of-the-art data-augmentation techniques, DeepSpectrumLite can produce powerful speech and audio analysis models with high accuracy that can then be easily deployed to embedded devices as an end-to-end prediction pipeline from raw microphone input. Our framework showed high performance for general speech-based paralinguistic tasks, music emotion recognition, and a range of speech and audio-based health monitoring tasks.

We have publicly released the framework including a flexible command-line interface on GitHub, such that interested researchers can use it in their own research for a variety of low-resource, real-time speech and audio recognition tasks, train their own models and apply them on embedded devices. Using the provided repository and the given parameters it is possible to reproduce all experiments conducted in this manuscript.

In Section 3.5, we have discussed the subject of explainability for our framework and given more insight into the decision making process of the DNN models by analysing the generated audio plots, DNN models' predictions, and confusion matrices. A quantitative study comparing Deep Spectrum against both audio pre-trained models and untrained CNNs has been undertaken in Amiriparian et al. (2020) demonstrating the efficacy of using image pre-trained CNNs for audio tasks. Motivated by the findings in Amiriparian et al. (2020), we have decided to use pre-trained image CNNs as deep feature extractors in our framework.

For future work, further reductions in model size can be pursued. From an efficiency standpoint of view, networks specifically designed with smaller memory and computation footprints than DenseNet121, such as Mobilenets (Howard et al., 2017) or SqueezeNet (Iandola et al., 2016) can be a better choice for the targeted applications and thus should be evaluated as feature extractors in DeepSpectrumLite . Using our framework, it is possible to select between 14 base models, including the above-mentioned CNNs. Furthermore, techniques such as pruning and quantization (Han et al., 2015; Lin et al., 2019; Zhou et al., 2019) can be explored together with their impacts on speed and model accuracy. Finally, future iterations of our framework could also be enhanced with explainability methods like those mentioned above to make the model predictions more interpretable.

## Data Availability Statement

The original contributions presented in the study are included in the article/supplementary material, further inquiries can be directed to the corresponding author/s.

## Author Contributions

SA, TH, VK, and MG conceptualized the study and ran the machine learning experiments. SO and BS gave technical advice, did literature analysis, manuscript preparation, and editing. MG and VK helped with running the experiments and testing the codes. All authors revised, developed, read, and approved the final manuscript.

## Funding

This study presented has received funding from the BMW Group. Further, we acknowledge funding from the DFG's Reinhart Koselleck project No. 442218748 (AUDI0NOMOUS) and the DFG project No. 421613952 (ParaStiChaD).

## Conflict of Interest

The authors declare that the research was conducted in the absence of any commercial or financial relationships that could be construed as a potential conflict of interest.

## Publisher's Note

All claims expressed in this article are solely those of the authors and do not necessarily represent those of their affiliated organizations, or those of the publisher, the editors and the reviewers. Any product that may be evaluated in this article, or claim that may be made by its manufacturer, is not guaranteed or endorsed by the publisher.

## Footnotes

^1^ https://github.com/DeepSpectrum/DeepSpectrum

^2^ https://github.com/DeepSpectrum/DeepSpectrumLite

^3^ https://www.tensorflow.org/lite
